# Observations on the Nature of Corns, and the Means of Removing Them

**Published:** 1797

**Authors:** Anthony Carlisle

**Affiliations:** Surgeon to the Westminster Hospital.


III. *Obſervations on the Nature of Corns, and the Means of removing them. Communicated in a Letter to Dr.* Simmons, *F. R. S. by Mr.* Anthony Carliſle, *Surgeon to the Weſtminſter Hoſpital.*

*To Dr.* SIMMONS.

SIR,

I TAKE the liberty of laying before you the following obſervations upon the nature and treatment of corns, and other ſimilar excreſcences of the cuticle, as a ſubject not unworthy the attention of the medical profeſſion; becauſe want of ſkill in the treatment of trifling diſeaſes is very juſtly an occaſion of reproach againſt against regular practitioners, and it alſo leaves a fair opening for empirics.

I have been the more deſirous to make theſe obſervations public, from having often found them uſeful to myſelf in practice; and I have alſo reaſon to ſuſpect that they are not generally known to medical men. In addition to theſe arguments, I hope that the ſucceeding remarks may be found the means of improving the cure of ſome painful and troubleſome diſeaſes, which although of little importance in the eſtimation of many perſons, yet are difficult to remedy without a previous knowledge of their nature from regular data.

The cuticular covering of the human body appears to be lifeleſs and inorganic * upon its outer ſurface. It is connected by veſſels to living parts upon its under ſurface, and is probably vaſcular when firſt formed. It is compoſed of lamina, of different degrees of thickneſs, and is ſuperficially divided into diſtinct maſſes of various forms in different parts of the body; for example, the ſcales of the cuti-

* By inorganic, I here mean deſtitute of that arrangement of veſſels, and connection with the acting parts of the body, which would otherwiſe have ſubjected it to the ſame conditions as the living parts.

cle which are ſhed from the legs are unlike thoſe from the palms of the hands.

There are holes through the cuticle for the matter of perſpiration to paſs out, and other openings for the tranſmiſſion of hairs. The former are ſimilar to the holes through the inorganic ſhell of an egg, and do not appear to be under the influence of life; the veſſels leading to theſe holes are, however, ſuſceptible of various changes. It would ſeem that the proceſſes of the cuticle which paſs into theſe holes in the true ſkin, form an open canal between the exhalent artery and the outward air. It does not appear that this external covering of the body is at all acted upon by the abſorbing ſyſtem of veſſels.

The cuticle is capable of undergoing many changes whilſt it is attached to the living body, without its being afterwards thrown off. It may be diſtended with water to a very unnatural thickneſs, as on the hands of perſons working in warm water; but will recover its former ſtate again in a few hours, after leaving off the uſe of the water.

The cuticle may be ſcorched with fire, or coagulated, ſo that it never reſumes its natural appearance appearance again, and yet it ſhall continue to cleave to the living body.

The cuticle ſeems alſo capable of putrefaction whilſt upon the living body.

In perſons who are liable to much perſpiration about their feet, and who are at the ſame time not in the habit of waſhing them often, I have many times obſerved the thick cuticle upon the under ſurfaces of the toes, and at their junction with the ſole of the foot, in a very putrid ſtate, diſſolving and rotting until the cutis itſelf was expoſed ſo much as to bleed.

The putrefaction of this ſubſtance is accompanied with a peculiar ſmell, and not the matter of perſpiration, as is vulgarly ſuppoſed.

The connection between the living body and the cuticle may be deſtroyed by a variety of ſtimulating applications, and even particular ſtates of the body produce untimely ſeparation of this covering; for example, œdema, ſome fevers, violent courſes of mercury, &c. When the ſeparation of the cuticle from the living body is produced rapidly, a quantity of the ſerum of the blood is thrown out between it and the living parts, as in the caſes of ſcalds, burns, bliſters, &c. This is a curious ſyſtem of defence. When the ſeparation of the cuticle is effected more flowly, it fometimes happens that the new layer of cuticle is formed before the old one lofes its hold, and in this way the fubftances of the two will be interwoven together; the accumulation of fucceffive layers clinging together in this manner forms a corn. The application of moderate preffure feems to be conducive both to the growth of new cuticle, and to the adhefion of the new with the old.

A corn will often originate from a fmall blifter, produced by fevere preffure, more efpecially when the blifter is accompanied with much inflammation of the fkin beneath. In this cafe the coagulating lymph is thrown out into the blifter, and becomes the bafis of a corn.

The natural procefs is for the old cuticle to be continually fhed in the form of fcales as foon as the new is formed.

When the cuticle is fuddenly removed from the living parts, as in the cafes of blifters, &c. before mentioned, the veffels of the fkin throw out a quantity of the ferum of the blood; they alfo throw out the coagulating lymph, which appears to be the fubftance from which the cuticle is formed. When the parts beneath the cuticle are effentially injured by the ftimulus which feparated the cuticle, it often follows that the lymph is thrown out very irregularly, forming ridges in ſome places, and unnaturally thin coverings in others. Theſe phenomena appear to depend upon the unequal actions of the living parts below. A ſtrong ſtimulating application laid over the whole extent of the ſore is a remedy for this.

The cuticle, when firſt formed, is of a curdly cheeſe-like ſubſtance, and the thickneſs of each layer depends upon the quantity of this ſubſtance thrown out at one time, which is very irregular.

The natural cuticle is a maſk of the ſurfaces of the living body to which it is attached.

The forming of cuticle upon a naked living ſurface, appears to be haſtened by the application of ſome ſubſtances which coagulate the animal mucilages, ſuch as æther and alcohol. Stimulating the living parts is alſo often neceſſary, in order to produce that degree of action in the veſſels which attends the throwing out of the coagulating lymph. The ſubſtance of the cuticle may be diſſolved by the application of Cauſtic Alkali, or *Aqua Kali pur.*

The moſt uſual occaſion of corns is the application of moderate and long-continued preſſure. In this caſe a proceſs is ſet up to defend the the living ſenſible parts againſt the injury, by thickening the inſenſible covering or cuticle. In this inſtance, however, the natural means made uſe of do not anſwer the end, and the reaſon might be given. The preſſure is commonly made at firſt upon a ſmall ſurface; the thickening of the cuticle increaſes this preſſure, by diminiſhing the ſpace between the preſſing ſubſtance and the living part. Still, however, new layers of cuticle are formed, and the true ſkin begins to be removed out of the way by abſorption, thus allowing the diſeaſed maſs of cuticle to ſink below the level of the living parts; proceeding upon this plan a cone of cuticle is formed with its apex protruded among ſenſible ſubſtances, ſo that upon preſſing or moving the part conſiderable pain enſues.

The difference of the forms of theſe bodies has given riſe to a variety of vulgar names, which it is not neceſſary to enumerate, becauſe theſe obſervations are intended to apply to every poſſible ſpecies of the diſeaſe, upon natural principles. There are, however, two kinds of corns, which differ in many reſpects from each other; the one is called the hard, the other the ſoft corn; the latter is always ſituated in a per-

ſpiring part, which ſeems to be the occaſion of its peculiarity.

It muſt be underſtood, that although the ſubſtance of a corn is compoſed of the ſame kind of matter as the cuticle, yet it is not modelled in the ſame manner; the layers are thicker, are often cheeſy in parts, and generally admit of the evaporation of a part of the water naturally combined with them; ſo that they form hard bodies, much more brittle and inflexible than the natural cuticle.

Sometimes inflammation takes place in the ſkin at the root of a corn, and the diſeaſed cuticle is thus diſlodged by a ſmall abſceſs: this is the natural cure.

I thought it right to premiſe thus much of the natural hiſtory of this part of the body, in order that the principles upon which every remedy to be hereafter adviſed, may be clearly underſtood. The diſeaſed ſtate of this ſubſtance is not always radically cured without ſome trouble, and different means muſt be applied in different caſes, ſuitable to the circumſtances of each. The matter of the cuticle itſelf, which forms this diſeaſe, is a ſubſtance of little conſequence; but in certain habits of body the extreme parts, where corns are uſually to be found, found, are often much injured, both by the diſeaſe and by injudicious attempts to remedy it; the extreme parts of the body being, in general, leſs under the command of medical treatment than the other parts of the body.

There are many rational methods of curing the diſeaſe called corns. The moſt common is by cutting or paring them; this never anſwers any farther purpoſe than temporary relief, becauſe the remaining cuticle is ſo diſeaſed, that it does not recover the power of ſhedding the old external lamina, whilſt new layers are forming underneath; and from this cauſe the corn will be ſoon renewed. Another objection to cutting is the impoſſibility of completely paring the corn when its under ſurface is conical or irregular, ſo as to leave only one layer of cuticle remaining, which ought to be done, elſe the corn will grow again. In ſome particular corns, however, the connection between the diſeaſed ſubſtance and the ſkin is ſo ſlight, that a line may be perceived where they unite, and with a blunt pointed inſtrument the corn may be ſeparated and completely turned out; but this is only in ſome old corns.

One method of curing corns radically is by diſſolving them. After having ſoftened them by ſteeping the part in warm water, apply the liquid cauſtic alkali, or *aqua kali pur.* upon a piece of ſponge, keeping it on about a quarter of an hour, or until a little pain is felt. A portion, if not all the corn, will be ſo diſſolved by this fluid, as to be eaſily rubbed off with a coarſe cloth, and this proceſs muſt be repeated until all the diſeaſed cuticle is removed. The management of this application requires ſome addreſs, and often conſiderable patience and perſeverance; ſo that many people are not diſpoſed to adopt it; and, beſides, if it be not followed up until the cutis itſelf is naked, there is no certainty of our having entirely deſtroyed the diſeaſed ſtructure.

In the above manner, hard and thick nails, which are producing inconvenience, may be alſo ſoftened, and the troubleſome part removed.

Another method of cure, which is, however, far more tedious and inconvenient, is by the application of pieces of adheſive plaſter, ſpread upon leather, each piece having a round hole cut in it, ſo as to admit the corn to paſs through. In this manner pieces of the plaſter are placed over each other, until they riſe above the level of the top of the corn; and a ſhoe is to be worn of a bigneſs ſufficient to contain the whole,

ſo

ſo that the preſſure ſhall fall, by means of theſe plaſters, upon the ſkin immediately ſurrounding the baſes of the corns. The conſequence of this is the protruſion of the ſunk root of the corn, and it is ſhed like natural cuticle. It requires that theſe plaſters be kept conſtantly applied for the ſpace of from three to ſix weeks, before they anſwer the purpoſe. Very adheſive plaſter, ſpread upon firm buckſkin leather, ſeems to ſuit this end exceedingly well. This method is always effectual, if judiciouſly applied and perſevered in; and it is not attended with any hazard of ill conſequences.

A third method of curing corns is by the application of a bliſter. The corn ſhould be firſt pared as cloſe as poſſible, and a piece of bliſtering plaſter applied, big enough to cover the baſe of the corn, and to paſs a little way over the ſound cuticle. The bliſter requires, in general, to remain upon the part for twenty-four hours, and ſhould be continued longer, provided it neither produces veſication nor pain. The common bliſtering plaſter is not ſtrong enough; it requires the addition of a little Euphorbium. If much inflammation attends, it may often be removed by the application of a ſimple milk and bread poultice. This method is particularly objectionable in old perſons, and in bad conſtitutions, becauſe violent irritation produced in the extremities of the body, in ſuch caſes, does not always terminate well.

The method of cure by bliſtering is, however, one of the moſt effectual and expeditious, and perhaps the only one for the ſoft corns.

At the concluſion of theſe obſervations, it may be neceſſary to remark, that I have ſpoken of the effects of all the foregoing remedies, as they have fallen under my own experience; but it is no more than juſt to add, that much of the reaſoning, as well as the practice, was ſuggeſted by the late Mr. John Hunter, and that the whole plan is founded on principles adopted in the Hunterian ſchool.

